# *Ec*CXCR4b influences RGNNV proliferation by interacting with the RGNNV capsid protein

**DOI:** 10.1128/mbio.02045-25

**Published:** 2025-11-12

**Authors:** Xiaojing Hua, Chen Li, Yanzhi Yu, Yuanan Lu, Jing Ye, Xueqin Liu

**Affiliations:** 1 National Key Laboratory of Agricultural Microbiology, College of Fisheries, Huazhong Agricultural University 47895, Wuhan, Hubei, China; 2 Hubei Engineering Technology Research Center for Aquatic Animal Diseases Control and Prevention, Wuhan, Hubei, China; 3 Department of Public Health Sciences, University of Hawaii at Manoa, Honolulu, Hawaii, USA; 4 National Key Laboratory of Agricultural Microbiology, College of Veterinary Medicine, Huazhong Agricultural University 47895, Wuhan, Hubei, China; Johns Hopkins University Bloomberg School of Public Health, Baltimore, Maryland, USA

**Keywords:** RGNNV, capsid protein, *Ec*CXCR4b, interaction protein, internalization

## Abstract

Red-spotted grouper nervous necrosis virus (RGNNV) is an intensely virulent pathogen affecting cultured fish, capable of infecting various reared marine and freshwater species worldwide and causing severe financial losses in the aquaculture business. The capsid protein (CP) of RGNNV, as the viral surface’s sole structural protein, is essential for mediating RGNNV binding to susceptible host cells. However, the cellular receptors involved in RGNNV entry are still mostly unknown. In this study, immunoprecipitation-mass spectrometry (IP-MS) was employed to screen for candidate proteins that could interact with RGNNV-CP. The *Epinephelus coioides* CXC chemokine receptor 4b (*Ec*CXCR4b) was found to be primarily localized on the cell membrane of grouper spleen (GS) cells and was identified as an interaction protein of RGNNV-CP by immunoprecipitation and immunofluorescence analysis. Furthermore, overexpression of *Ec*CXCR4b in host cells enhanced RGNNV attachment and internalization, whereas silencing *Ec*CXCR4b expression significantly inhibited replication of RGNNV and reduced viral entry. Additionally, treating cells with anti-CXCR4 antibodies to block cell surface *Ec*CXCR4b, or pretreating RGNNV virions with recombinant *Ec*CXCR4b protein effectively inhibited RGNNV binding to GS cells. *In vivo,* administering recombinant *Ec*CXCR4b protein significantly increased the survival rate of RGNNV-infected groupers. Moreover, exogenous overexpression of *Ec*CXCR4b facilitated RGNNV internalization in typically non-susceptible human lung adenocarcinoma cells (A549). Notably, we discovered that the clathrin endocytosis pathway was the mechanism by which *Ec*CXCR4b mediated RGNNV entrance into host cells. The results suggest that *Ec*CXCR4b is essential for RGNNV entry into host cells and could potentially serve as a therapeutic target for interventions against RGNNV infections.

**IMPORTANCE:**

Red-spotted grouper nervous necrosis virus (RGNNV) is the most prevalent genotype in Betanodavirus and infects numerous fish species in warm water environments. In the viral infectious life cycle, virus-receptor recognition and interactions play a crucial role. Nevertheless, the host interaction proteins involved in RGNNV infection are still not completely understood. Therefore, further research is required to identify additional host proteins involved in RGNNV invasion to refine our understanding of its pathogenic mechanisms. In this study, we identified *Epinephelus coioides* CXC chemokine receptor 4b (*Ec*CXCR4b) as RGNNV-CP interacting protein, and further analyses revealed that this interaction is of vital importance for RGNNV attachment and internalization. Additionally, *Ec*CXCR4b facilitates RGNNV entry into host cells via the clathrin-dependent endocytic mechanisms. These findings suggest that *Ec*CXCR4b serves as a critical host factor involved in RGNNV endocytosis, providing new insight into the intricate mechanisms underlying RGNNV entrance and useful prospective targets for antiviral medication development.

## INTRODUCTION

Nervous necrosis virus (NNV) is a small, non-enveloped, icosahedral RNA virus belonging to the *Nodaviridae* family and classified as *Betanodavirus* (1). NNV acts as the pathogenic agent of viral encephalopathy and retinopathy, which is commonly referred to as viral nervous necrosis. This virus primarily infects post-hatch larvae, fingerlings, as well as juvenile fish, leading to exceptionally high mortality rates and considerable financial losses for the aquaculture industry (2, 3). The viral genome is constituted by two positive-sense, single-stranded RNA segments, RNA1 and RNA2; along with subgenomic RNA3, which is situated in the 3′-terminal domain of RNA1. These elements encode RNA-dependent RNA polymerase (RdRp), the structural capsid protein (CP), as well as non-structural B1 and B2 proteins of the virus, respectively (4). The overall topology of the CP includes the N-terminal ARM (N-ARM, 99 bp), the N-terminal arm (N-arm, 54 bp), the shell domain (S-domain, 486 bp), the linker region (LR, 21 bp), and the protrusion domain (P-domain, 354 bp) (4). The variable region RNA2 T4 is used in a phylogenetic analysis to classify bethanodaviruses into four genotypes (5). These genotypes include tiger puffer nervous necrosis virus, striped jack nervous necrosis virus, barfin flounder nervous necrosis virus, and red-spotted grouper nervous necrosis virus (RGNNV). Among them, RGNNV is the most prevalent genotype, as it affects numerous fish species in warm water environments, and the 25–30°C is the ideal temperature for its growth (6).

As intracellular pathogens, viruses utilize one or multiple receptors to attach to the host cells and penetrate the plasma membrane, enabling their infectious life cycle (7, 8). Virus-receptor recognition and interactions play a crucial regulatory role in determining host range, tissue tropism, and viral pathogenesis (7, 9). The identification of viral receptors and understanding of their interaction mechanisms provide valuable insights for developing novel antiviral therapies and vaccine technologies (9, 10). As the sole structural protein displayed on the virus particle’s surface, the CP participates in the viral invasion and encapsidation during betanodavirus infection (2, 11). Previous research has demonstrated that CP determines host specificity (12). Several studies have explored the interaction between CP and host proteins in facilitating viral infection. Notably, the receptors GHSC70 and *Mm*HSP90ab1 have been successfully characterized, which facilitate RGNNV attachment and entry into host cells (13, 14); Nectin1 plays a critical role in RGNNV entrance by directly interacting with CP to anchor unbound virions and manage viral internalization (15). Similarly, Nectin4 has been shown to interact with CP and may serve as an NNV entry receptor (16). A recent study identifies marine medaka MYL3 as a novel receptor for RGNNV and shows it mediates viral entry via macropinocytosis through the IGF1R-Rac1/Cdc42 axis (17). Additionally, *Ec*TRAF4 and *Ec*AnxA2 have been identified as co-localizing with CP in the cytoplasm, where they promote RGNNV infection by inhibiting IFN responses (18, 19). RGNNV infection involves intricate interactions between the virus and various host proteins, many of which remain poorly unidentified. Therefore, further research is required to identify additional host proteins involved in RGNNV invasion, ultimately refining our understanding of its pathogenic mechanisms.

Chemokine receptors, as a family of seven-transmembrane (TM) domain G protein-coupled receptors (GPCRs), are commonly divided into CC chemokine receptors (CCRs), CXCRs, XCRs, and CX3CRs depending on the kind of chemokine they trap (20, 21). CXCR4 stands out as the most extensively studied CXCR member, owing to its crucial roles in various biological processes, including development, immunological response, and illness. It exerts its functions by binding to its primary ligand, CXCL12 (alternatively termed stromal-derived factor 1) (22, 23). The CXCR4 receptor is characterized by a structural organization comprising seven hydrophobic TM domains, three extracellular loops (ECLs), three intracellular loops, an extracellular N-terminus, and an intracellular C-terminus (24). It has been recognized to play a significant role in various human illnesses, such as cancer, autoimmune disorders, and immunodeficiencies (25). Notably, CXCR4 functions as a co-receptor that mediates the cellular entrance of human immunodeficiency virus (HIV) and simian immunodeficiency virus (SIV), enabling these viruses to infect host cells (26, 27). During Epstein-Barr virus (EBV) infection, the maintenance of EBV latency is facilitated by the interaction between the EBV-encoded products LMP2A and CXCR4 (28). Additionally, CXCR4 and ligand CXCL12 show elevated expression in the immunological tissues of teleost fishes when the host is invaded by viruses or bacteria, suggesting that the CXCR4/CXCL12 axis is essential to the fish’s innate immune system (24, 29–31). Lin et al. were the first to clone *Epinephelus coioides* CXCR4 and investigated its expression profile in response to NNV infection in 2012 (30). Their findings indicated that CXCR4 is involved in the immune system’s initial reaction to pathogenic microorganisms. However, the specific mechanism of CXCR4’s role in viral infections remains to be completely clarified.

In this study, we employed immunoprecipitation-mass spectrometry (IP-MS) to identify *Epinephelus coioides* CXC chemokine receptor 4b (*Ec*CXCR4b) as an RGNNV-CP interacting protein. Further analyses revealed that this interaction is of vital importance for RGNNV attachment and internalization. Additionally, *Ec*CXCR4b facilitates RGNNV entry into host cells via the clathrin-dependent endocytic mechanisms. Overall, our findings suggest that *Ec*CXCR4b serves as a critical host factor involved in RGNNV endocytosis and represents a potential target for antiviral strategies against RGNNV infection.

## RESULTS

### *Ec*CXCR4b interacts with RGNNV-CP

The CP, as the only structural protein of betanodavirus, controls host specificity in the viral infection process (2, 12). To identify the possible RGNNV CP-interacting host proteins, grouper spleen (GS) cells were used as a vulnerable cell line and infected with RGNNV. The co-immunoprecipitation (Co-IP) assay was performed utilizing the resin, which had been immobilized with anti-RGNNV-CP mAb at 24 h post-infection. Subsequently, the immunoprecipitated proteins were identified as multiple unique proteins from the experiment group by liquid chromatography-tandem mass spectrometry (Table S1). *Ec*CXCR4b, as a potential CP-interacting protein, was discovered and initially investigated for its role in RGNNV infection.

**Fig 1:**
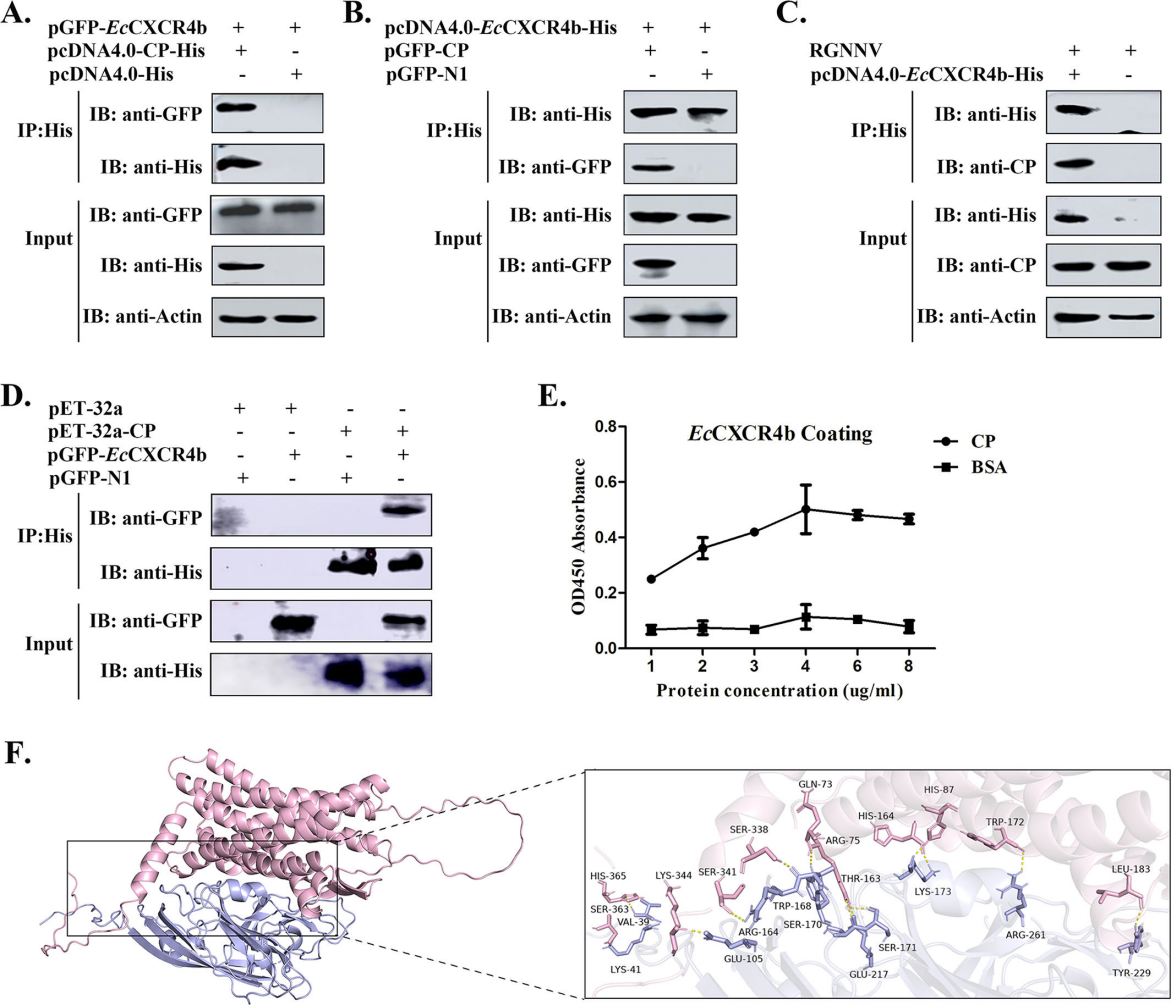
*Ec*CXCR4b interacts with RGNNV-CP. (**A–C**) Co-IP assay was performed to verify the interaction between *Ec*CXCR4b and CP. GS cells were co-transfected with plasmid combinations indicated in the figures or RGNNV-infected GS cells, which were transfected with pcDNA4.0-*Ec*CXCR4b-6His. After being collected, the cell lysates were immunoprecipitated using anti-His Abs. (**D**) His pull-down of His-CP with *Ec*CXCR4b *in vitro*. Lysates from GS cells overexpressing pGFP-*Ec*CXCR4b were subjected to pull-down with the specified His fusion proteins. Both pull-down complexes and lysates were immunoblotted with the specified antibodies. (**E**) ELISA was adopted to analyze the biomolecular interactions of *Ec*CXCR4b and RGNNV-CP. (**F**) Molecular docking model of *Ec*CXCR4b with RGNNV-CP. The amino acid residues of CP interacting with *Ec*CXCR4b are indicated (right panel).

First, a Co-IP assay was performed to verify the interaction between CP and *Ec*CXCR4b. As illustrated in Fig. 1A through C, *Ec*CXCR4b co-precipitated with the CP in an IP assay when epithelioma papulosum cyprini (EPC) cells that were co-transfected with pGFP-*Ec*CXCR4b/pcDNA4.0-CP-6His or pGFP-CP/pcDNA4.0-*Ec*CXCR4b-6His plasmids or RGNNV-infected GS cells, which were transfected with pcDNA4.0-*Ec*CXCR4b-6His, were treated with mAb directed against His. Also, a His tag pull-down experiment verified the direct interaction between RGNNV-CP and *Ec*CXCR4b (Fig. 1D). Furthermore, this result was confirmed by ELISA experiments using purified proteins of His-*Ec*CXCR4b and His-Cp (Fig. 1E). Finally, the protein interaction between *Ec*CXCR4b (pink) and CP (blue) was predicted by HADDOCK24 and visualized by Pymol (Fig. 1F), and the docking simulation model showed that CP interacts with *Ec*CXCR4b displayed in pink sticks (right panel). Taken together, these results indicated that *Ec*CXCR4b interacted with the RGNNV-CP.

### *Ec*CXCR4b localizes on the cell surface

**Fig 2:**
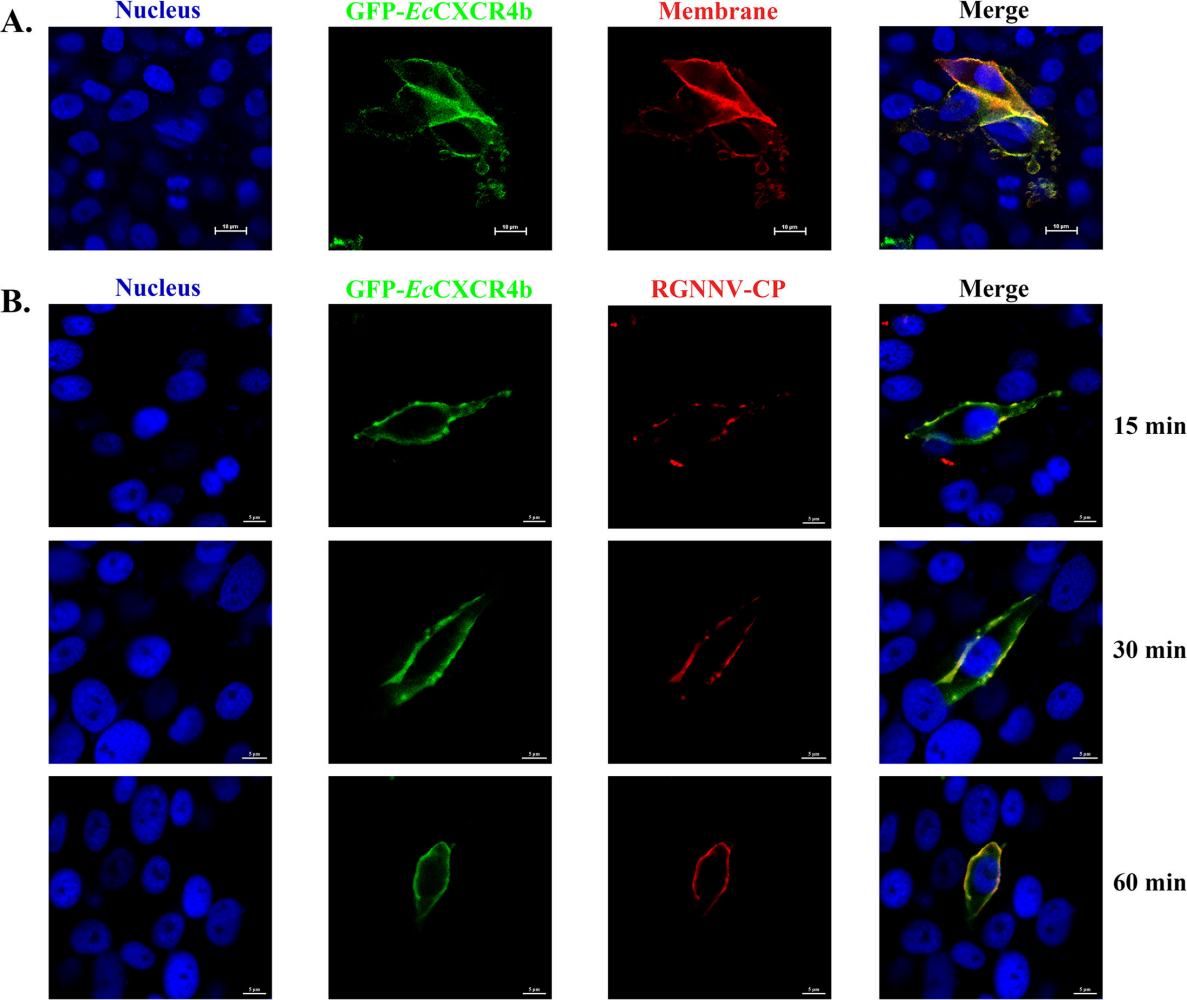
*Ec*CXCR4b localizes on the cell surface. (**A**) The intracellular localization of *Ec*CXCR4b. GS cells were transfected with the pGFP-*Ec*CXCR4b plasmid, and the nuclei and cell membrane were stained with DAPI and Dil, respectively. (**B**) Co-localization of endogenous RGNNV-CP and *Ec*CXCR4b in GS cells at different exposure times. The GFP-*Ec*CXCR4b-transfected GS cells were infected with RGNNV at an MOI of 10 for 15, 30, and 60 minutes at 4°C, fixed with formalin, and immunostained with anti-RGNNV CP Abs. The fluorescent signal was observed and captured with a confocal laser scanning microscope.

*Ec*CXCR4b belongs to the GPCR superfamily; it is widely understood that human GPCRs are seven-TM proteins (32). To explore whether *Ec*CXCR4b of grouper distributes on the surface of GS cells and contributes to RGNNV infection, first, the subcellular localization of *Ec*CXCR4b was analyzed by observing the expression of the objective protein with GFP tag. The plasmid of pGFP-*Ec*CXCR4b was transfected into GS cells, and the fluorescent signal was monitored through a confocal laser scanning microscope. The result, as shown in Fig. 2A, was indicated by the cell membrane’s red fluorescent signal, and the green fluorescence of GFP-*Ec*CXCR4b proteins was primarily distributed on the membrane. Subsequently, the GFP-*Ec*CXCR4b-transfected GS cells were exposed to virus (multiplicity of infection [MOI] = 10) or L-15 basic medium for 15, 30, or 60 minutes at 4°C. The cellular localizations of *Ec*CXCR4b and RGNNV-CP were analyzed using a fluorescence microscope. As shown in Fig. 2B, *Ec*CXCR4b and CP protein colocalized on the cell membrane, and the number of RGNNV virions increased with longer exposure times of 15–60 minutes. These findings indicated that *Ec*CXCR4b localized on the surface of cells and assisted virions in attaching to cell surfaces during RGNNV infection.

### ECL2 of *Ec*CXCR4b is the crucial domain for interacting with the LR domain of CP

**Fig 3:**
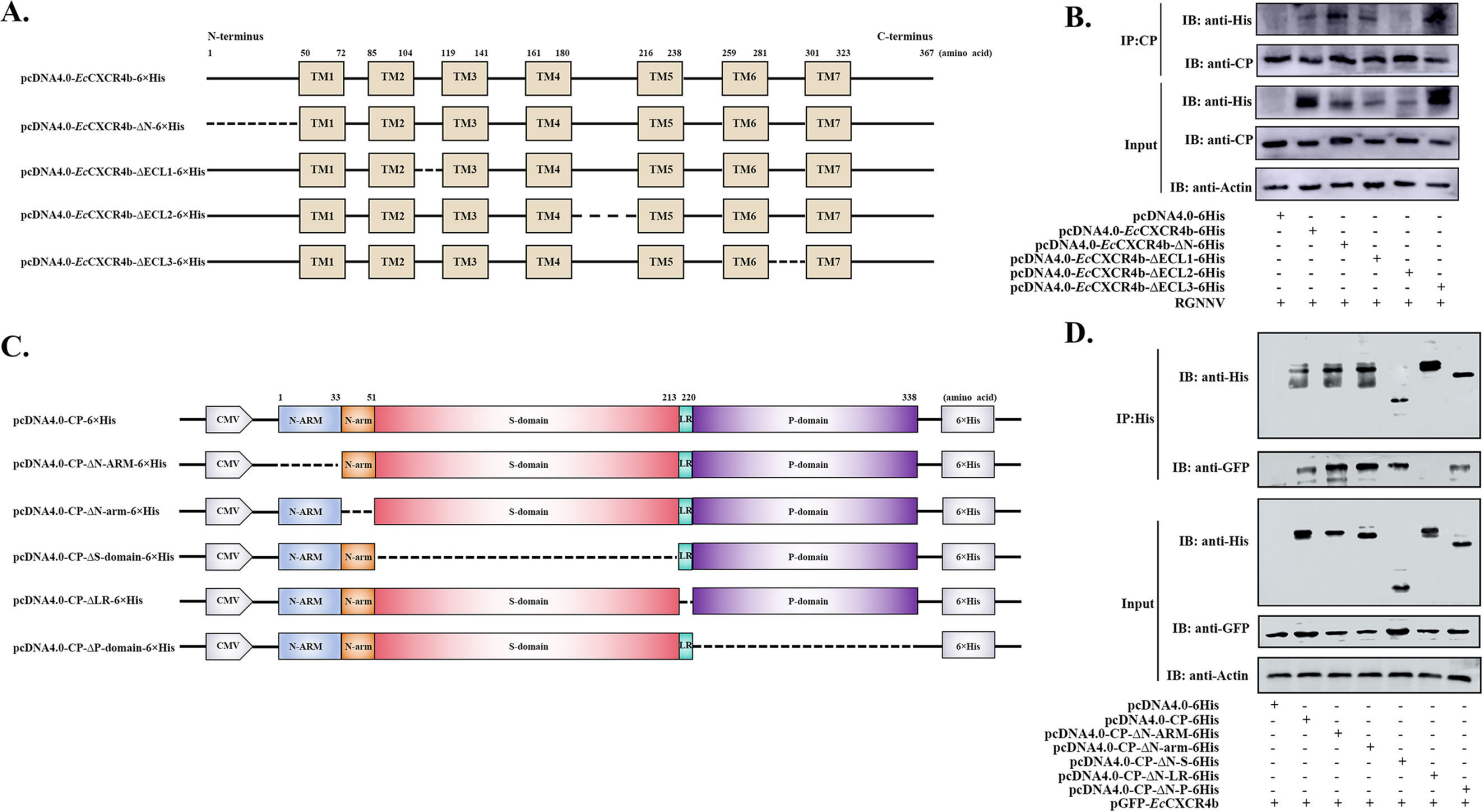
*Ec*CXCR4b interacts with the LR domain of RGNNV-CP structure via ECL2. (**A**) Schematic illustration of the full-length *Ec*CXCR4b and its truncated forms for the Co-IP analysis. (**B**) Co-IP assays were carried out to detect the key ECL of *Ec*CXCR4b. GS cells were transfected with the plasmid combinations indicated in the figures. After 24 h, the GS cells were allowed natural contact with the virus at 4°C to conduct the virus binding assay. Following, the cells were lysed, and the Co-IP assay was performed using anti-RGNNV-CP mAb. The precipitated complexes and input samples were immunoblotted using the specified antibodies. (**C**) RGNNV-CP full-length and truncated forms are shown schematically for the CO-IP analysis. (**D**) Co-IP experiments were performed to identify the critical domain of the CP protein. GS cells were co-transfected with the plasmid combinations depicted in the diagram. After 24 h, cells were lysed, and Co-IP was conducted using anti-His antibodies, and the precipitated complexes and input samples were analyzed by immunoblotting with the appropriate antibodies.

Based on the structural localization of CXCR4b to the cell membrane according to previous reports (24), five eukaryotic expression plasmids were constructed to express ECL domain-deficient *Ec*CXCR4b mutants (Fig. 3A). To identify the critical ECLs of *Ec*CXCR4b that catch RGNNV particles during virus infection, GS cells were transfected with the different recombinant plasmids to simulate natural receptors and permitted to naturally engage with RGNNV at 4°C. Figure 3B illustrates that exogenous expression of the truncated *Ec*CXCR4b could capture a number of free RGNNV particles for attachment to the cell surface, except for the deficient mutant of *Ec*CXCR4b-ΔECL2. The result indicated that the ECL2 of *Ec*CXCR4b was the crucial domain for interacting with CP.

Moreover, to determine the key interaction domain of the CP protein with the *Ec*CXCR4b, we constructed 6× His-tagged deletion mutant plasmids of the CP fragments (Fig. 3C) and successfully expressed each deletion mutant’s recombinant protein. pGFP-*Ec*CXCR4b was transfected into GS cells together with the plasmids listed above, and Co-IP procedures were conducted for subsequent analysis. The results showed that GFP-*Ec*CXCR4b coprecipitated with His-ΔN-ARM, His-ΔN-arm, His-ΔS-domain, and His-ΔP-domain following treatment with anti-His antibody, except His-ΔLR (Fig. 3D), thus indicating that the LR of RGNNV-CP structure was the essential region for the interaction with *Ec*CXCR4b. Taken together, our data suggested that ECL2 of *Ec*CXCR4b is the crucial domain for interacting with the LR domain of CP.

### *Ec*CXCR4b is required for RGNNV infection

**Fig 4:**
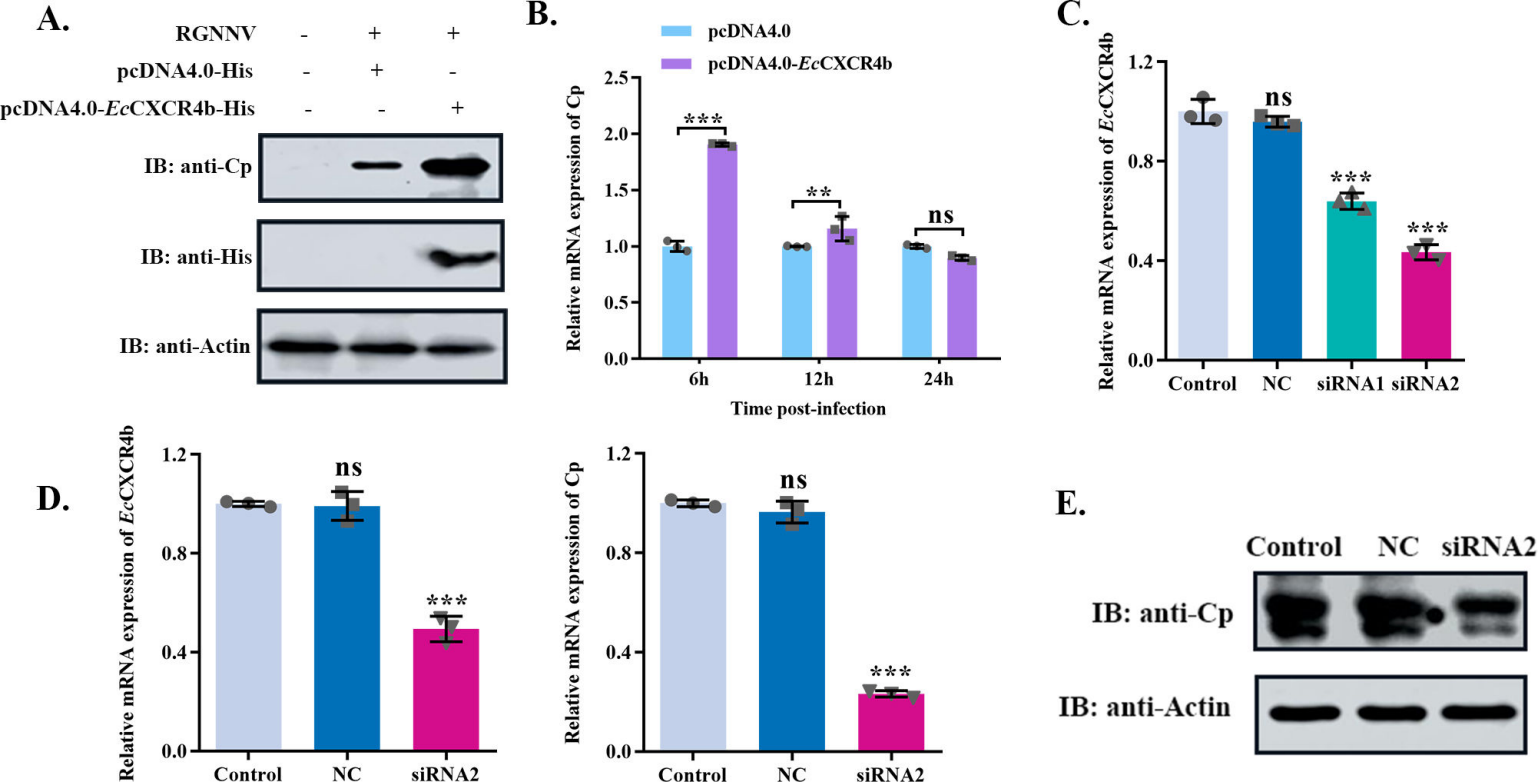
*Ec*CXCR4b is involved in RGNNV infection in GS cells. (**A and B**) Impact of *Ec*CXCR4b overexpression on virus replication. GS cells were transfected with pcDNA4.0-*Ec*CXCR4b-6His or pcDNA4.0-6His plasmids and infected with virus (MOI = 1). The infected cells were collected for western blotting (**A**) and qRT-PCR (**B**). (**C**) RNA interference on the expression of endogenous *Ec*CXCR4b. si*Ec*CXCR4b or control siRNA (NC) was transfected into GS cells. The efficiency of siRNAs targeting *Ec*CXCR4b was utilized by qRT-PCR. (**D and E**) *Ec*CXCR4b knockdown inhibited RGNNV infection. GS cells were transfected with siRNA2 or NC and infected with the virus (MOI = 1). The infected cells were harvested for qRT-PCR (**D**) and western blotting (**E**). Data represent means ± standard deviation. *P < 0.05, **P < 0.01, and ***P < 0.001.

To investigate the function of *Ec*CXCR4b in RGNNV infection, the *Ec*CXCR4b gene was overexpressed and knocked down in GS cells by transfected with pcDNA4.0-*Ec*CXCR4b-6His or specific siRNA, followed by incubation with virus for 24 h at 28°C, and subsequently the virion was monitored by quantitative real-time polymerase chain reaction (qRT-PCR) and western blotting. The data indicated that the overexpression of *Ec*CXCR4b in GS cells enhanced viral infection (Fig. 4A and B). By examining the relative mRNA expression of *Ec*CXCR4b, it was found that the transfection of siRNA2 resulted in a more efficient knockdown of *Ec*CXCR4b than siRNA1 (Fig. 4C). Next, GS cells were infected with the virus for 24 h after being transfected with siRNA2 or control siRNA (NC) for 24 h. Subsequent result analysis indicated that the knockdown of *Ec*CXCR4b effectively suppressed the relative mRNA expression and protein level of RGNNV-CP in GS cells (Fig. 4D and E). These results indicate that the knockdown of *E*cCXCR4b suppresses RGNNV proliferation.

### *Ec*CXCR4b is essential for RGNNV attachment and internalization

**Fig 5:**
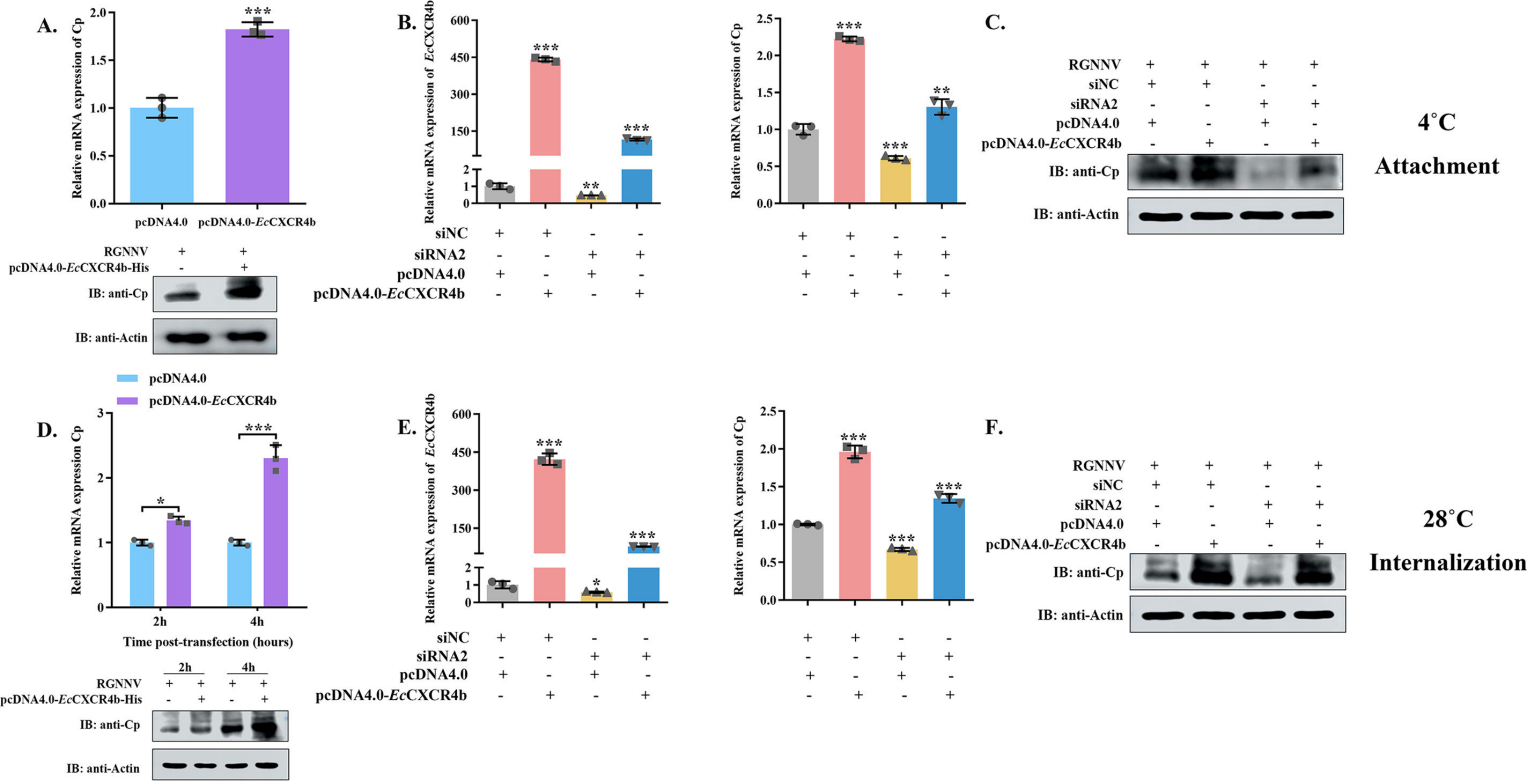
Role of *Ec*CXCR4b in RGNNV attachment and internalization. (**A and D**) Effect of overexpression of *Ec*CXCR4b on RGNNV attachment (**A**) and internalization (**D**). GS cells were transfected with pcDNA4.0-6His or pcDNA4.0-*Ec*CXCR4b-6His plasmids for 24 h, and then the cells were infected with the virus at different temperatures. RGNNV-CP expression levels were detected using qRT-PCR and western blotting. (**B–F**) Effect of knockdown of *Ec*CXCR4b expression on RGNNV attachment and internalization. GS cells were co-transfected with *Ec*CXCR4b siRNA2 or NC along with pcDNA4.0-6His or pcDNA4.0-*Ec*CXCR4b-6His plasmids for 24 h, respectively. Then, cells were infected with the virus for 2 h at 4°C (**B and C**) or 4 h at 28°C (**E and F**), and the expression level of RGNNV-CP was measured using qRT-PCR and western blotting. Data represent means ± standard deviation. *P < 0.05, **P < 0.01, and ***P < 0.001.

Viruses possess simple structure and composition features, whereas they engage in complex interactions with host cells, particularly during the entry stage (33). To evaluate the function of *Ec*CXCR4b during RGNNV entry, we first performed viral attachment and internalization assays. The *Ec*CXCR4b gene was over-expressed and knocked down in GS cells by transfecting with pcDNA4.0-*Ec*CXCR4b-6His or specific siRNA, followed by incubation with virus for 2 h or 4 h at different temperatures (4°C or 28°C), and the virion was detected by qRT-PCR and western blotting finally. At 4°C, the virus demonstrates the ability to bind to cells while failing to penetrate them (34). The results demonstrated that overexpressing *Ec*CXCR4b in GS cells enhanced viral attachment (Fig. 5A) and internalization ability (Fig. 5D). Conversely, si*Ec*CXCR4b-transfected cells showed a significant decrease in viral attachment (Fig. 5B and C) and internalization (Fig. 5E and F) compared to the NC-transfected group, and the opposite effect could be rescued by overexpression of *Ec*CXCR4b. Collectively, these results indicate that *Ec*CXCR4b is essential for RGNNV attachment and internalization.

### Recombinant *Ec*CXCR4b protein or CXCR4b antibody inhibits RGNNV infection *in vitro* and *in vivo*

**Fig 6:**
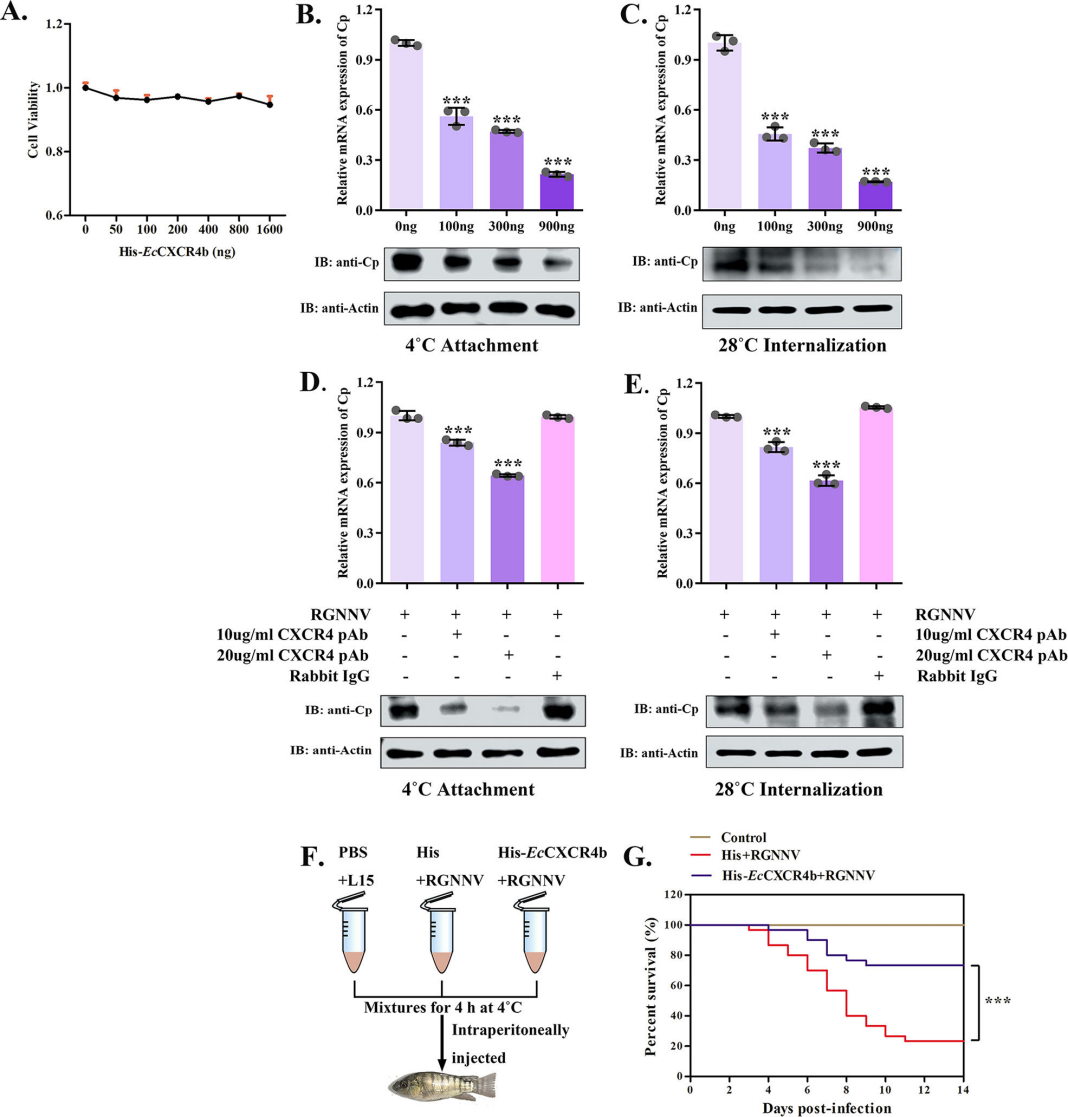
Recombinant *Ec*CXCR4b protein and an antibody against CXCR4b block RGNNV infection. (**A**) Toxicity assay for GS cellular effects of recombinant His-*Ec*CXCR4b protein at different concentrations. (**B and C**) Recombinant *Ec*CXCR4b protein blocks RGNNV infection. RGNNV was pre-incubated with the obtained His-*Ec*CXCR4b protein, then the mixtures were introduced to GS cells for another 2-h incubation period at 4°C (**B**) or 4 h at 28°C (**C**). (**D and E**) An antibody against CXCR4b blocks RGNNV infection. GS cells were blocked with anti-CXCR4b antibodies and exposed to virus for 2 h at 4°C (**D**) or 4 h at 28°C (**E**). The RGNN-CP expression was detected using qRT-PCR and western blotting. (**F and G**) Survival rates of groupers after intracerebrally injected with the mixtures of RGNNV and recombinant His-*Ec*CXCR4b or 6× His protein, the PBS and L15 medium mixtures as the negative control group. Data represent means ± standard deviation. *P < 0.05, **P < 0.01, and ***P < 0.001.

To further assess the function of cell membrane-localized *Ec*CXCR4b during RGNNV entry, a blocking assay was performed using purified His-*Ec*CXCR4b protein or anti-CXCR4b pAb. The purified His-*Ec*CXCR4b protein had no effect on cell survival at the various concentrations tested (Fig. 6A). RGNNV was preincubated with the purified protein, and then GS cells were treated with mixtures. Findings showed that purified His-*Ec*CXCR4b protein inhibited RGNNV virus infection in GS cells in a dose-dependent manner (Fig. 6B and C). Additionally, GS cells were preincubated with anti-CXCR4b pAb or IgG as control and then infected with RGNNV. qRT-PCR and western blotting results revealed a dose-dependent inhibition of viral attachment and entry (Fig. 6D and E). To validate the impact of *Ec*CXCR4b on RGNNV infection *in vivo*, the groupers received intraperitoneal injections of His-*Ec*CXCR4b protein or 6× His and RGNNV mixtures, the PBS with L-15 medium mixture serving as the control group (Fig. 6F). The His-*Ec*CXCR4b group exhibited a survival rate of 73.3%, while the survival rate of 6× His was 23.3% (Fig. 6G). Accordingly, these findings implied that *Ec*CXCR4b played a role in RGNNV entry and that RGNNV infection might be prevented by preventing the interaction between RGNNV and *Ec*CXCR4b.

### *Ec*CXCR4b mediates the internalization of RGNNV in A549 cells

**Fig 7:**
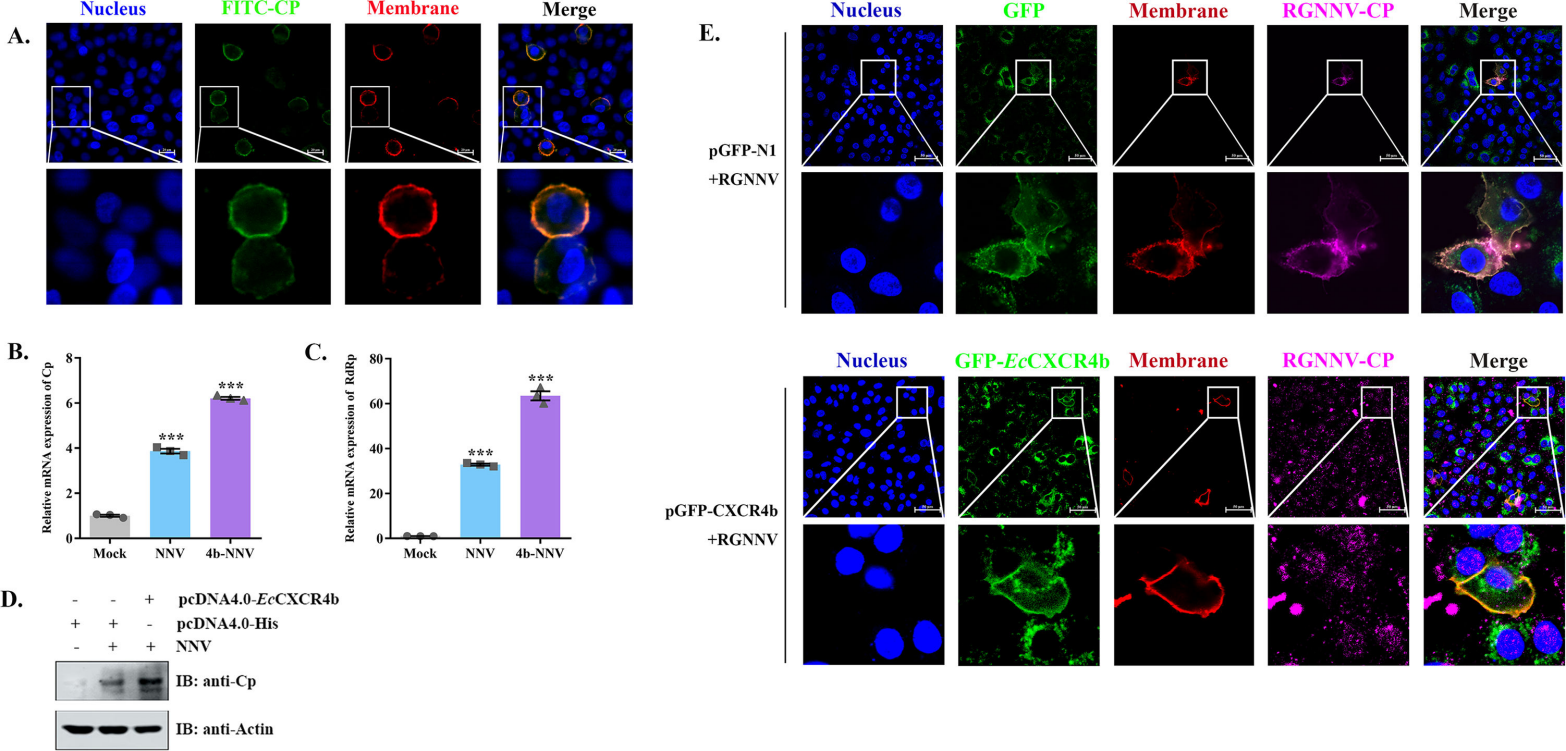
*Ec*CXCR4b empowers RGNNV particles’ entry into non-sensitive cells, A549. (**A**) Assessment of the co-localization of RGNNV particles only attached to the A549 cellular surface. A549 cells were incubated with RGNNV at an MOI of 10, and after 24 h, the cells were then subjected to immunofluorescence staining with anti-RGNNV CP Abs. The fluorescent signal was observed with a confocal laser scanning microscope (bar = 20 µm). (**B–D**) Effect of overexpression of *Ec*CXCR4b on RGNNV replication in A549 cells. A549 cells were transfected with pcDNA4.0-*Ec*CXCR4b-6His plasmids or pcDNA4.0-6His for 24 h, then incubated with virus (MOI = 10) for 24 h. RGNNV copies were measured through qRT-PCR (**B and C**) and western blotting (**D**). (**E**) A549 cells were transfected with pGFP-*Ec*CXCR4b or pGFP-N1 plasmids for 24 h, then incubated with virus (MOI = 10) for 24 h, followed by the A549 cells being treated as described above (bar = 50 µm). Data represent means ± standard deviation. *P < 0.05, **P < 0.01, and ***P < 0.001.

The attachment and internalization of viruses mainly depend on host factors on the target cell membrane (35). A previous study suggested that RGNNV has the capacity to infect human cell lines while demonstrating its binding to cells without penetration (36). To validate that *Ec*CXCR4b mediates the internalization of RGNNV on cells, A549 cells were used in this study. First, A549 cells were subjected to RGNNV infection (MOI = 10) for 24 h, and subcellular localization revealed that RGNNV-CP indeed only attached to the cellular membrane (Fig. 7A). Following transfection with either the control plasmid pEGFP-N1 or the recombinant plasmid pEGFP-*Ec*CXCR4b, A549 cells were infected with RGNNV 24 hours later. The qRT-PCR and western blotting results suggested that the overexpression of *Ec*CXCR4b promotes RGNNV infection in A549 cells (Fig. 7B through D). Subsequently, the intracellular immunofluorescence localization of CP further verified RGNNV internalization in *Ec*CXCR4b-transfected A4549 cells. Compared to the control plasmid pGFP-N1-transfected group, the pGFP-*Ec*CXCR4b-transfected group showed a large number of virion internalizations in A549 cells and observed several viral particles in the cytoplasm (Fig. 7E). The above results indicated that exogenous *Ec*CXCR4b played a role in mediating the internalization of RGNNV in A549 cells.

### *Ec*CXCR4b mediates RGNNV entry through the clathrin endocytosis pathway

**Fig 8:**
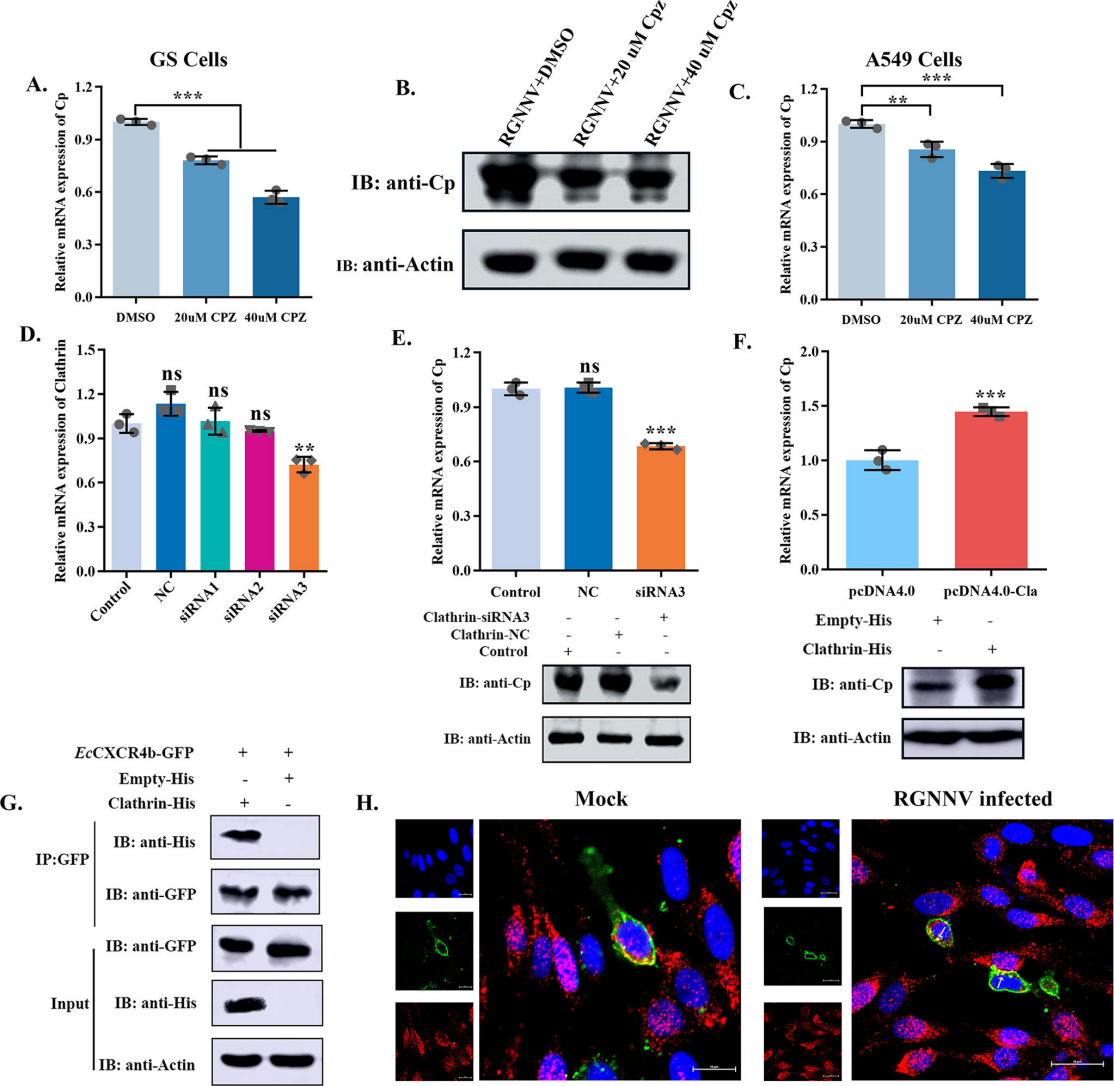
*Ec*CXCR4b mediates RGNNV entry through the clathrin endocytosis pathway. (**A and B**) Effect of clathrin endocytosis pathway inhibitor on viral internalization in GS cells. GS cells were subjected to pretreatment with various doses of CPZ before the virus infection. Then the cells were collected to detect the expression level of RGNNV-CP through qRT-PCR and western blotting. (**C**) Effect of clathrin endocytosis pathway inhibitor on viral internalization in A549 cells. The *Ec*CXCR4b-transfected A549 cells were also subjected to the same treatment as described above, and the CP expression level was analyzed using a qRT-PCR assay. (**D**) Knockdown of endogenous clathrin expression via RNA interference. siRNA for clathrin or control siRNA (NC) was transfected into GS cells. After 24 h, qRT-PCR was employed to evaluate the knockdown efficiency of siRNAs targeting clathrin expression. (**E and F**) Effect of expression of clathrin on RGNNV replication in GS cells. Clathrin expression in GS cells was either downregulated or upregulated, and then the internalization assays were performed. The expression of RGNNV-CP was determined using qRT-PCR and western blotting. (**G and H**) The interaction of *Ec*CXCR4b and RGNNV-CP was investigated by Co-IP and intracellular immunofluorescence localization assays. GS cells were co-transfected with plasmid combinations indicated in the figures for 24 h Western blotting was used to examine the cell lysates after they had been immunoprecipitated using anti-GFP Abs (**G**). GS cells were transfected with pGFP-*Ec*CXCR4b/pcDNA4.0-Clathrin-6His plasmids for 24 h, then one group was incubated with virus (MOI = 10) for 4 h at 28°C, another group was a mock. The cells were then subjected to immunofluorescence staining using a specified antibody, and cell nuclei were stained with DAPI. The fluorescent signal was observed with a confocal laser scanning microscopy (bar = 20 µm) (**H**). Data represent means ± standard deviation. *P < 0.05, **P < 0.01, and ***P < 0.001.

Ligand stimulation induces CXCR4 endocytosis, which has been reported to be endocytosed by a clathrin-dependent pathway (37). Previous research has reported that clathrin-mediated endocytosis (CME) is one of the routes by which RGNNV enters the host cell (33). Therefore, we hypothesized that *Ec*CXCR4b is involved in RGNNV endocytosis via the clathrin-mediated pathway. First, chlorpromazine (CPZ), as a clathrin endocytosis pathway inhibitor, was used; GS cells were pretreated with CPZ and subsequently infected with the virus (MOI = 10). The results showed that CPZ treatment at different safety concentrations exhibited an inhibitory effect on RGNNV internalization (Fig. 8A and B). Moreover, further research indicated that CPZ also attenuated *Ec*CXCR4b-mediated RGNNV internalization in *Ec*CXCR4b-transfected A549 cells (Fig. 8C). To validate our observations, RGNNV infection ability was examined when using pcDNA4.0-clathrin-6His or siRNA to overexpress or silence the target clathrin gene, respectively. The clathrin-expressing efficiency was confirmed by qRT-PCR (Fig. 8D), resulting in opposite expression levels of RGNNV-CP (Fig. 8E and F). In addition, the interaction of *Ec*CXCR4b and RGNNV-CP was examined by Co-IP and intracellular immunofluorescence localization (Fig. 8G and H). Collectively, these findings indicate that *Ec*CXCR4b facilitates RGNNV entering GS cells through the CME pathway.

## DISCUSSION

More than 120 fish species are susceptible to NNV, a highly fatal and contagious virus that primarily infects larvae and juvenile fish, significantly hindering the global aquaculture industry (1, 38, 39). Since many aspects of NNV biology and pathogenesis remain unknown, substantial systematic research efforts are required to mitigate the damage caused by this disease. The viral life cycle consists of several phases: entry, genome replication, protein synthesis, assembly, and release from the host cell (13). In these steps, the early interaction between the virions and cellular surface receptors represents a crucial step, serving as a prerequisite for viral internalization and effective infection (8). Thus, the identification of NNV receptors and elucidation of their interactions with the virus is essential for understanding viral pathogenesis. To date, there are several receptors that have been identified in RGNNV attachment or entry (13–17). Despite the involvement of various host proteins in RGNNV infection, the complexity of these interactions remains poorly understood. In this study, we employed IP-MS to screen for and identify a protein that localized on the cell membrane as a novel host component implicated in RGNNV infection.

The chemokine system plays a crucial part in the host’s immune response to combat microbial pathogens and in bridging innate and adaptive immunity (40). CXCR4, a member of the seven-TM domain GPCR family, has been identified as a co-receptor for HIV and SIV during the viral entry into host cells (27, 28). CXCR4 exhibits primarily expression in the central nervous system (CNS) and immunological organs. In grouper, gCXCR4 has been found to exhibit high expression in NNV target organs like the brain and eyes, as well as major lymphoid organs, including gills, spleen, and head kidney (30). Based on our screening results, we hypothesized that the *Ec*CXCR4b functions as a receptor associated with NNV infection and plays an essential role in the viral invasion process. Here, we utilized CO-IP, pull-down, and ELISA assays to confirm the interaction between *Ec*CXCR4b and CP. Furthermore, this interaction was observed in NNV-infected cells, indicating *Ec*CXCR4b may participate in RGNNV infection. Structurally, based on the distinct regions of CP and *Ec*CXCR4b described previously (4, 24), our findings demonstrate that the LR domain of CP is essential for its interaction with *Ec*CXCR4b-ECL2. Notably, suppressing *Ec*CXCR4b expression in GS cells significantly impeded RGNNV infection. These results highlight the essential function of *Ec*CXCR4b in the RGNNV infection process. Additionally, it is widely recognized that this virus genus is neurotropic and causes encephalopathy (6). Currently, accumulating evidence has demonstrated that certain neurotropic viruses, such as rabies virus, herpes simplex virus, and enterovirus 71 (EV71), can utilize the retrograde axonal transport mechanism to spread from peripheral nerve terminals to the CNS (41); thus, we speculate RGNNV may target CNS neurons via retrograde axonal transport or neuron-expressed *Ec*CXCR4b homologs. This further emphasizes that *Ec*CXCR4b is a key factor in RGNNV infection and may be associated with the pathways by which the virus targets CNS neurons.

The attachment and internalization of viruses primarily depend on host factors present on the target cell membrane (35). In this study, silencing *Ec*CXCR4b expression hindered RGNNV attachment and internalization, whereas overexpressing *Ec*CXCR4b had the opposite effect. Further analysis revealed that administering an *Ec*CXCR4b antibody or soluble His-*Ec*CXCR4b effectively blocked RGNNV attachment and internalization in a dose-dependent manner. Furthermore, animal experiments demonstrated that His-*Ec*CXCR4b protein provided partial protection against RGNNV infection in grouper. Notably, *Ec*CXCR4b reconstruction enabled substantial RGNNV invasion into inherently non-permissible cells, indicating *Ec*CXCR4b as a crucial invasion factor. Overall, these findings indicate *Ec*CXCR4b as a critical factor in both RGNNV attachment and internalization processes, supporting its identification as a novel functional receptor for infection with RGNNV. The role of CXCR4 in various diseases, including cancer, autoimmunity, and immunodeficiencies, has been well-documented (25). However, most studies have focused on the CXCR4/CXCL12 signaling axis in mammals and teleost fishes (23, 42–44). While the CXCR4/CXCL12 pathway exerts somewhat distinct functions across different organisms, an overarching trend of regulating immune responses remains consistent (44). For example, CXCR4/CXCL12 activation stimulates downstream signaling pathways such as MAPK3 and JAK/STAT, promoting antiviral response in gibel carp (24). Additionally, CXCR4/CXCL12 expression is upregulated following NNV or *Singapore grouper iridovirus* infection, suggesting a role in defending against viral invasion (30, 45). These findings appear to contradict the function of CXCR4 as a receptor for viral entry. However, this discrepancy may be attributed to differing perspectives—explaining its role from the standpoint of the host versus the virus. The CXCR4/CXCL12 axis is targeted by numerous pathogens, which use various strategies to modify or exploit CXCR4 activity. And some viruses could utilize it for cell entry or modulate its expression/functional activity—directly affecting cell trafficking, immune responses, proliferation, and survival (32). Thus, the dual nature of CXCR4 as both a viral receptor and a chemokine receptor not only presents a biological paradox but also a potential new therapeutic strategy for combating viral diseases.

The engagement of the appropriate receptor can lead to direct viral fusion at the plasma membrane or trigger various endocytic pathways, facilitating fusion within intracellular vesicular compartments best suited for the viral life cycle (46). Understanding viral entry pathways is crucial for elucidating viral pathogenesis and developing effective drugs to prevent infection at the earliest and critical stage (47). CME is a well-characterized endocytic pathway through which extracellular substances are transported into cells, and it serves as a primary entry route for both enveloped and nonenveloped viruses (46). Multiple studies have revealed that the CME pathway serves as the primary entry route for RGNNV into host cells (33). Additionally, CME serves as a major internalization pathway for CXCR4 and other chemokine receptors (37). Our findings show that treatment with a CPZ inhibitor and *Ec*CXCR4b-specific siRNA effectively inhibited CME and RGNNV entry into cells. Notably, CO-IP analysis revealed an interaction between *Ec*CXCR4b and clathrin protein in GS cells, suggesting that CP protein binds to *Ec*CXCR4b and subsequently facilitates RGNNV internalization via the CME pathway.

**Fig 9:**
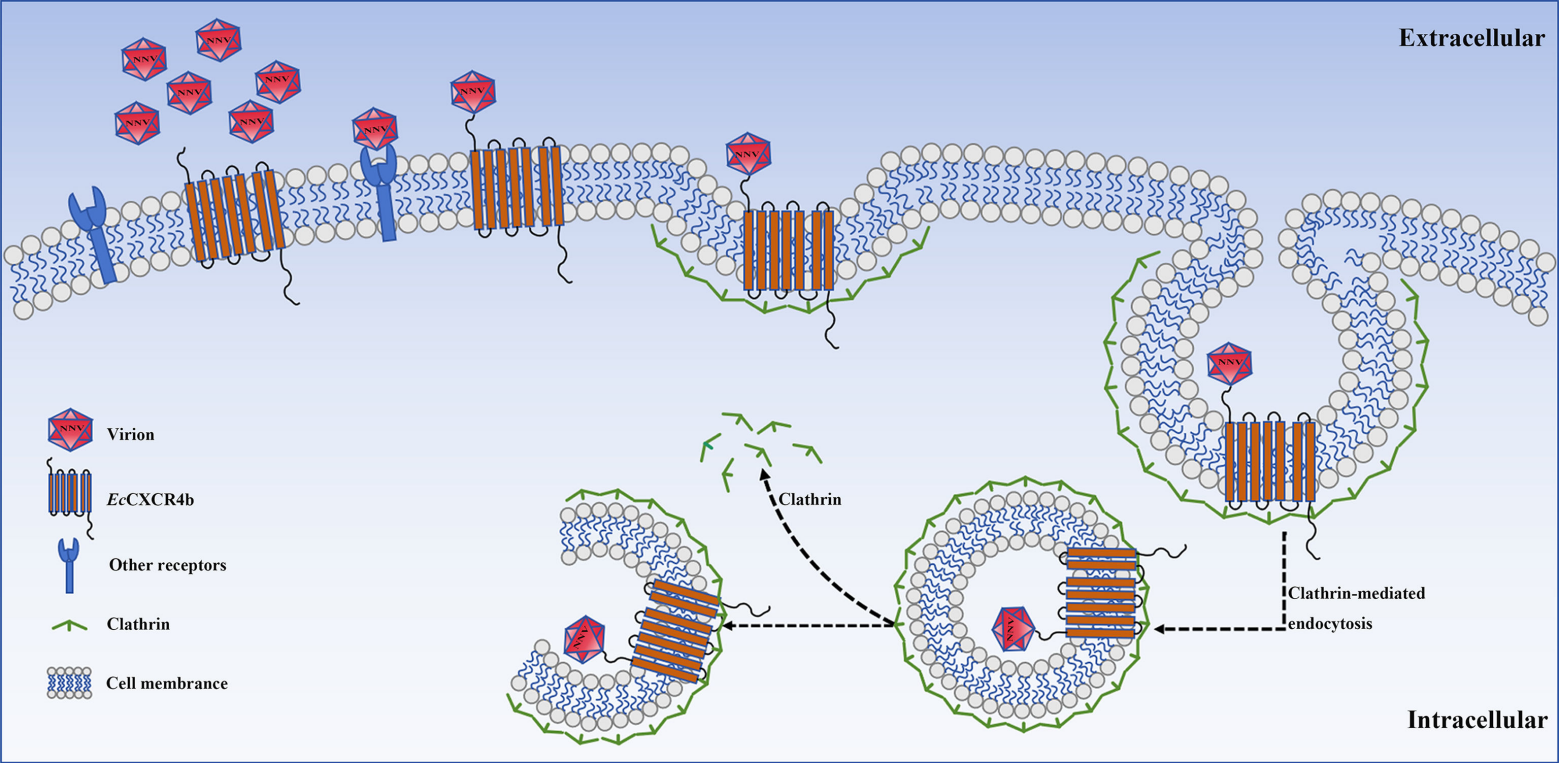
Model of the role of EcCXCR4b in RGNNV internalization. First, RGNNV attaches to the GS cell surface by binding to *Ec*CXCR4b through CP or other receptors. Then, the RGNNV, *Ec*CXCR4b, and clathrin compose a protein complex. Eventually, the virus particle is effectively internalized through the CME pathway.

In conclusion, our findings identify *Ec*CXCR4b as an interacting protein of RGNNV-CP and a key entry factor for RGNNV infection. *Ec*CXCR4b is localized on the cell membrane and plays an essential function in RGNNV attachment and internalization. Further analysis revealed that *Ec*CXCR4b mediates RGNNV entry into cells via the CME pathway (Fig. 9). These results deepen our understanding of the complex functions of CXCR4b in teleosts, provide novel insights into RGNNV pathogenesis, and offer new perspectives for managing RGNNV infections.

## MATERIALS AND METHODS

### Cells and viruses

GS cells were kindly gifted by Prof. Qiwei Qin (South China Agricultural University) and maintained in L-15 medium (Biosharp, China) supplemented with 10% fetal bovine serum (FBS, Biochannel) at 28°C and 5% CO_2_ (48). EPC cells were kept in our laboratory for preservation and cultured in Medium 199 (M199, Biosharp, China), supplemented with 10% FBS at a 28°C incubator. Human lung adenocarcinoma cells (A549) purchased from Servicebio (Wuhan, China) were grown in Ham’s F-12K medium (Servicebio, China) containing 10% FBS and 1% penicillin-streptomycin (PS), and A549 cells were kept at 37°C and 5% CO_2_. The virus of RGNNV was obtained from grouper and preserved by our laboratory (49). RGNNV production was carried out in GS cells and harvested through three freeze-thaw cycles.

### Plasmids, antibodies, and reagents

The CXCR4b open reading frame was amplified by PCR with specific primers designed according to the *Ec*CXCR4b reference sequence (MH716018.1). Then, the *Ec*CXCR4b fragment was cloned into pGFP-N1, pcDNA4/myc-6His A, and pET-32a vectors to obtain eukaryotic expression plasmids for transfection of cells and prokaryotic expression plasmid for protein purification, respectively. The plasmids used to express RGNNV-CP (MN309752.1) were constructed and stored in our laboratory (50, 51). The primers are tabulated in Table 1, and DNA sequencing was performed to analyze and identify all plasmids constructed in this study.

**TABLE 1:** Primer details of PCR and qRT-PCR experiment in this study (restriction enzyme sites for plasmid construction are underlined)

Primer name	Sequence(5′−3′)	Accession no.	Used
P4-EcCXCR4b-F	CGGAATTCGCCACCATGTCGTACTATGAGCATATC	MH716018.1	Plasmid construction
P4-EcCXCR4b-R	GCTCTAGAGCTGGAATGTAAACTGGAGG		
pGFP-EcCXCR4b-F	CCAAGCTTGCCACCATGTCGTACTATGAGCATA	MH716018.1	Plasmid construction
pGFP-EcCXCR4b-R	GGGGTACCCTAGCTGGAATGTAAACTGG		
pet32a-EcCXCR4b-F	CGGAATTCATGTCGTACTATGAGCATATCG	MH716018.1	Plasmid construction
pet32a-EcCXCR4b-r	CCAAGCTTGCTGGAATGTAAACTGGAG		
P4-Clathrin-F	CGGAATTCGCCACCATGGCTCAGATACTGCCGATACG	XM050050794.1	Plasmid construction
P4-Clathrin-R	GCTCTAGACATGTTGAAGCCATAAGCAGG		
Red-Cp-F	CCAAGCTTCGATGGTACGCAAAGGTGAGAAG	MN309752.1	Plasmid construction
Red-Cp-R	GGGGTACCTTAGTTTTCCGAGTCAACCC		
β-Actin-F	CTGACTCTGAAGTACCCCATCGA	JF430611.1	qRT-PCR
β-Actin-R	CGGAAGCGTACAGGGACAGC		
qEcCXCR4b-F	CACCTGCGTTGGTGTGAAC	MH716018.1	qRT-PCR
qEcCXCR4b-R	CATAAACCAGTTTGTGTGCCAG		
qCP-F	CCAACTGACAACGACCACAC	MN309752.1	qRT-PCR
qCP-R	CAGTACTGACACGTTGACCAC		
qRdRp-F	GTGTCCGGAGAGGTTAAGGATG	JN189882.1	qRT-PCR
qRdRp-R	CTTGAATTGATCAACGGTGAACA		

Rabbit anti-β-actin monoclonal antibody (mAb) (AC026), rabbit anti-CXCR4 polyclonal antibody (pAb) (A1303), horseradish peroxidase (HRP)-conjugated goat anti-rabbit IgG (H + L) (AS014), HRP-conjugated goat anti-mouse IgG (H + L) (AS003), Alexa Fluor 594-conjugated goat anti-mouse IgG (H + L) (AS054), Alexa Fluor 647-conjugated goat anti-mouse IgG (H + L) (AS059), and FITC-conjugated goat anti-mouse IgG (H + L) (AS001) were procured from ABclonal (Wuhan, China). Mouse anti-GFP mAb (66002-1-Ig) and mouse anti-His mAb (66005-1-Ig) were obtained from Proteintech (Wuhan, China); mouse anti-RGNNV-CP mAb was prepared and preserved in our laboratory (51). Dil (cell membrane red fluorescent probe, C1036), His-tag Protein Purification Kit (Denaturant-resistant, P2229S), and TMB horseradish peroxidase color development solution (P0209) were purchased from Beyotime (Shanghai, China). Enhanced cell counting Kit-8 (BL1055C) and DAPI staining solution (BL105A) were purchased from Biosharp (Hefei, China). Pierce Co-IP Kit used in this study (26149) was procured from Thermo Fisher Scientific (Waltham, MA, USA).

### Overexpression and RNA interference

GS or A549 cells were inoculated into 24-well plates or T75 cm^2^ dishes and cultured overnight, and the target plasmids or small interfering RNAs (siRNAs) were diluted with Opti-MEM (Gibco). After the cell confluence reached 80%, the mixed suspension was transfected into cells using QuickShuttle-Enhanced from Biodragon (Suzhou, China) by the manufacturer’s protocol. Gene expression profiles in cellular systems were confirmed through western blot analysis or quantitative PCR (qPCR) detection. The siRNA (Table S2) oligonucleotides used in the work were commercially synthesized by Sangon Biotech (Shanghai, China).

### RGNNV infection

Confluent GS or A49 cells were plated in the 12-well plates and incubated overnight. In experiments involving overexpression or siRNA, cells were infected with virus (MOI = 1) and incubated for 2 h at 28°C, followed by a prewarmed 28°C maintain medium containing 5% FBS added to the cells. Thereafter, the treated cells in the plate were subsequently cultured at 28°C and collected at scheduled times by cell scraping. To conduct the virus binding assay, the target cells were treated with the virus (MOI = 10) for 2  h at 4°C, which allowed the virus to attach to the cells’ surface without entering (34). Subsequently, the collected cell samples were rinsed three times with pre-cold phosphate-buffered saline (PBS) and lysed to detect the mRNA levels using qRT-PCR and protein levels using western blotting. To perform the viral internalization experiment, the cells were exposed to the virus (MOI = 10) for 2 h at 4°C and then were washed three times with pre-cold PBS to eliminate free virions, followed by a culture temperature shift to 28°C for 2 h or 4 h to allow for internalization. Then the externalized virions were eliminated by using an alkaline high-salt solution (50 mM NaHCO_3_, 1 M NaCl). Thereafter, the cells were lysed to perform qRT-PCR and western blotting tests for analyzing the target gene expression level in the cells. To collect the viral mRNA, the cells were lysed using multiple freeze-thaw cycles and then underwent a gradient centrifugation to remove cellular debris. The total RNA was extracted from the collected cell samples using an RNA extraction solution (G3013, Servicebio, China) according to the manufacturer’s instructions.

### Co-immunoprecipitation

Co-IP procedures were carried out using the Pierce Co-IP Kit following the manufacturer’s instructions. In T75 cm^2^ flasks, 20 µg of multiple plasmid combinations as stated were co-transfected into GS cells; after 24 h of transfection, the harvested cells were lysed with pre-cold IP lysis/wash buffer. Afterward, the cell lysis solution was collected through centrifugation for 10 min at 13,000 *× g* and added to the resin, which was immobilized with specific antibodies, and the mixture was incubated with gentle mixing or rocking overnight at 4°C. The immunoprecipitants were generated with an adequate amount of elution buffer and analyzed using western blotting.

### His-tagged fusion protein purification

To confirm the interaction between the RGNNVP-CP and *Ec*CXCR4b *in vitro*, the corresponding His-tagged CP and *Ec*CXCR4b prokaryotic proteins were generated in *E. coli*. In short, the pET-32a-CP and pET-32a-*Ec*CXCR4b plasmids were used to transform the *E. coli* strain BL21. Bacterial cultures were cultivated at 37°C for 12 h, and the following day, cultures were transferred to fresh LB medium at a 1:100 ratio and grown to an OD_600_ of 0.5–0.7. Next, cells were induced with 1 mM isopropyl-β-d-thiogalactopyranoside (IPTG) at 37°C for 4–6 h to ensure protein expression. For His-tagged protein purification, the medium was centrifuged at 4,000 *× g* for 20 min at 4°C to harvest the cell pellets. The fusion proteins were purified with a His-tag Protein Purification Kit (Denaturant-resistant, Beyotime), by the manufacturer’s instructions.

### Enzyme-linked immunosorbent assay

Enzyme-linked immunosorbent (ELISA) was also adopted as a widely used method in examining biomolecular interactions. In this study, the obtained His-*Ec*CXCR4b proteins were serially diluted to different concentrations in PBS and used to coat 96-well plates (100 µL/well) overnight at 4°C. The wells were blocked using 2% bovine serum albumin (BSA) in PBST at 37°C for 2 h after being washed thrice with PBST (PBS containing 0.1% Tween-20). The purified His-CP protein was dispensed into the microtiter plate and incubated for 2 h, with BSA serving as the negative control. Subsequently, the wells were rinsed thrice with PBST to eliminate non-binding protein. The primary antibody of mouse anti-His (1:500) was applied at 37°C for 2 h, followed by a secondary antibody of HRP-conjugated goat anti-mouse IgG (1:5,000) at 37°C for 1 h. Lastly, the TMB chromogenic solution was added to each well, and the reaction was halted using 2M H_2_SO_4_, and the OD_450_ absorbance value was read using a microplate reader.

### Pull-down assays

The His-CP fusion protein was co-incubated with GFP-*Ec*CXCR4b or GFP-vector protein, which were extracted from GS cell lysates following transfection with pGFP-*Ec*CXCR4b or empty pGFP-N1 empty vectors, respectively (the control groups in this experiment included three combinations: pGFP-N1/pET-32a-CP, pGFP-EcCXCR4b/pET-32a, and pGFP-N1/pET-32a). The lysate prepared from GS cells was subjected to centrifugation to minimize background interference in subsequent detection or analysis procedures. The concentration of the target protein was determined prior to the experiment to quantify protein concentration, ensuring a standardized amount of protein was added to each reaction system. After that, the immunoprecipitated complexes were enriched by resins, which were immobilized with His antibodies to perform standard pull-down assays at 4°C overnight. The resins were washed three times with IP lysis/wash buffer to eliminate an unconjugated mixture of the bait and prey proteins, and elution buffer was added to collect the flow-through, which was subjected to immunoblot analysis.

### Immunofluorescence and confocal microscopy studies

GS cells were plated in a glass-bottomed size 15 mm confocal dish and transfected with the specified plasmids or infected with the virus. Following 24 h incubation, the cellular monolayer was washed thrice with PBS before fixing in 4% paraformaldehyde for 30 minutes. The cells were then treated with a specific primary antibody for 1 hour at room temperature after being blocked for one hour with 2% BSA in PBS. Following three rounds of PBST washing, the cells were treated for an hour with the indicated fluorophore-conjugated secondary antibody. The cellular nuclei and membrane were stained with DAPI and Dil, respectively. All fluorescence images were observed and acquired with a confocal laser scanning microscope N-STORM (Nikon, Japan). And a 40× objective was used to capture the panoramic view of cell monolayers, facilitating the selection of representative imaging areas. The primary antibodies used are as follows: mouse anti-RGNNV-CP mAb (1:1,000) and mouse anti-His mAb (1:500). The secondary antibodies include the following: Alexa Fluor 594-conjugated goat anti-mouse IgG (H + L) (1:200), Alexa Fluor 647-conjugated goat anti-mouse IgG (H + L) (1:200), and FITC-conjugated goat anti-mouse IgG (H + L) (1:500).

### Blocking assays

The above purified *Ec*CXCR4b proteins with different concentrations (100, 300, and 900 ng) were incubated with RGNNV at 4°C for 4 h. GS cells were cultured in 12-well plates until 90% confluence and subsequently treated with virus and protein mixtures. The cells were treated with the same procedures described above to test the impact of the recombinant protein on the virus-infected host cells.

A rabbit anti-CXCR4 pAb was used for the antibody-blocking assay, which possesses high homology between humans and fish, and isotype antibody IgG was used as control. GS cells were plated in 24-well plates and cultured for 12 h to allow for adhesion, followed by treatment with antibody suspension (10 and 20 µg/mL) and incubated for 4 h at 28°C. After this, the cells were washed three times with L15 medium and infected with RGNNV (MOI = 10) according to the method described above to monitor the amount of virus binding and internalization.

### Survival assay

Healthy experimental fish, with a sex ratio of 1:1 (male to female), were procured from a grouper farm in Guangzhou, China, and the average body weight was 1.22 ± 0.04 g (⁓4 cm). The experimental fish were maintained in freshwater at 25°C and randomly assigned to three groups: the PBS group, the His group, and the His-*Ec*CXCR4b group (*n* = 30). Viral suspensions of RGNNV (10^5^ TCID_50_/mL/fish) were first incubated for 4 hours at 4°C with either the recombinant His-*Ec*CXCR4b protein or His-tag control protein, followed by separate intraperitoneal injection into groupers. The negative control group of fish received the same volume of PBS and L15 medium mixtures. The daily number of fish deaths in three groups was recorded for 14 days, and each group’s survival rate was calculated.

### Drug inhibition assays

CPZ was used to inhibit CME. GS and A549 cells were subjected to different concentrations of CPZ (20 µM and 40 µM) for 2 h before RGNNV infection. Next, the cells were infected with RGNNV (MOI = 10) for 2 h at 4°C and then washed thrice with pre-cold PBS to remove the free virions, and the culture temperature was raised to 28°C for 4 hours to allow for internalization. Then, the not-yet-internalized virions were eliminated by utilizing the alkaline high-salt solution. After that, the cells were lysed to examine the internalized virions in the cells using the qRT-PCR and western blotting assays.

### Western blotting

The immunoprecipitants and whole-cell lysate samples were separated by SDS-PAGE gel, and then proteins were transferred to polyvinylidene difluoride (PVDF) membranes. The PVDF membrane was blocked for 1 h with 2% BSA at room temperature before being treated with the particularized primary antibody for 2 h. Then, the membrane was washed three times with PBST and incubated with the HRP-conjugated secondary antibody. Following three times washes with PBST buffer, the target immunoblot was visualized using the chemiluminescent substrate reagent and imaged using a chemiluminescence detection system.

### qRT-PCR assay

Cell samples were collected using RNA extraction solution (Servicebio, China) for the total RNA extraction, referring to the manufacturer’s suggested procedure. A reverse transcription kit (ABclonal, China) was employed to produce the cDNAs. To detect the target genes’ mRNA expression level, the qRT-PCR was performed using the 2 × Universal SYBR Green Fast qPCR Mix (ABclonal, China). The relative mRNA expression levels of genes from three independent biological replicates were measured by the 2^−∆∆Ct^ method with β-actin mRNA serving as an endogenous control. All the primers for qRT-PCR are listed in Table 1.

### IP-MS detection and data analysis

GS cells were infected with RGNNV (MOI = 1) or mock-infected and then lysed using the pre-cold IP lysis/wash buffer provided in the Pierce Co-IP Kit. The cell lysate was centrifuged at 13,000 *× g* and 4°C for 10 min, and the supernatant was incubated with the resin, which was immobilized with RGNNV-CP mAb at 4°C overnight. After washing three times with IP lysis/wash buffer, the dried beaded agarose resin was sent to SpecAlly Life Technology (Wuhan, China) for dynamic interaction omics mass spectrometry analysis. Mass spectrometry analysis was performed on a trapped ion mobility quadrupole time-of-flight mass spectrometer timsTOF Pro (Bruker Daltonics). An UltiMate 3000 RSLCnano system (Thermo) was coupled online to the timsTOF Pro with a CaptiveSpray nano ion source (Bruker Daltonics).

MS raw data were analyzed with MaxQuant (V1.6.6) using the Andromeda database search algorithm. Spectrum files were searched against the UniProt database using the following parameters: LFQ mode was checked for quantification; variable modifications, oxidation (M), acetyl (Protein N-term) & deamidation (NQ); fixed modifications, carbamidomethyl (C); digestion, trypsin/P; The MS1 match tolerance was set as 20 ppm for the first search and 4.5 ppm for the main search; the MS2 tolerance was set as 20 ppm. Search results were filtered with 1% false discovery rate at both protein and peptide levels. Proteins denoted as decoy hits, contaminants, or only identified by sites were removed, and the remaining identifications were used for further quantification analysis. The missing values were imputed from a random Gaussian-shaped distribution around the detection limit of the mass spectrometer, which applied a downshift of 1.8 times the standard deviations and a width of 0.25 times the standard deviations. Valid value filtering was performed with at least 50% of valid values in at least one group. In this study, proteins with a fold change > 2 between bait IP and control were screened out as interactors of the bait protein.

### Animal ethics statements

The fish experimental protocol was carried out in compliance with the guidelines of the Animal Experimental Ethical Committee on Animal Research at Huazhong Agricultural University, China (ID Number: HZAUFI-2025-0051). For minimizing fish suffering, 3-aminobenzoic acid ethyl ester methane sulfonate (MS-222) (Sigma, USA) was utilized to anesthetize all fish during the infection experiment.

### Statistical analysis

All values in this work were shown as means ± standard deviation from three independent biological replicates. Student’s *t*-test was used for comparisons between two groups, and one-way analysis of variance (ANOVA) followed by Tukey’s multiple comparisons test was used for comparisons of multiple groups. GraphPad Prism software was used for statistical calculations and data plotting. Differences in means were considered statistically significant at *P*  <  0.05, and significance levels are as follows: *P  <  0.05; **P  <  0.01, and ***P  <  0.001.

## Data Availability

All data are available in the main text or the supplemental materials. And the MS raw data have been deposited to the ProteomeXchange Consortium (https://proteomecentral.proteomexchange.org) via the iProX partner repository with the data set identifier PXD068633.

## SUPPLEMENTAL MATERIAL

Table S1 mbio.02045-25-s0001.docx Analysis and screening of candidate proteins by immunoprecipitation-mass spectrometry (IP-MS).

Table S2 mbio.02045-25-s0002.docx siRNA sequence details of transfection experiment in this study.

ASM does not own the copyrights to Supplemental Material that may be linked to, or accessed through, an article. The authors have granted ASM a non-exclusive, world-wide license to publish the Supplemental Material files. Please contact the corresponding author directly for reuse.
