# Supplementary material for: EcCXCR4b influences RGNNV proliferation by interacting with the RGNNV capsid protein
Source: mBio. 2025 Nov 12;16(12):e02045-25. doi: 10.1128/mbio.02045-25 (PMC12691606; doi:10.1128/mbio.02045-25)
Supplement: Table S1 — Analysis and screening of candidate proteins by immunoprecipitation-mass spectrometry (IP-MS). [file mbio.02045-25-s0001.docx]

**S1 Table Analysis and screening of candidate proteins by immunoprecipitation-mass spectrometry (IP-MS)**

| Number | **Accession** | **Gene** | **Description** | **Mw(kDa)** | **Length** | **Fold Change** |
| --- | --- | --- | --- | --- | --- | --- |
| 1 | U5TUM5 | ND5 | NADH-ubiquinone oxidoreductase chain 5 | 67.672 | 612 | 4.585132237 |
| 2 | U5TUM0 | ATP6 | ATP synthase subunit a (Fragment) | 25.031 | 227 | 2.778395433 |
| 3 | G0XMT3 | cxcr4b | C-X-C chemokine receptor type 4 protein | 40.127 | 367 | 2.535045717 |
| 4 | D7R610 | CPT1a | Carnitine O-palmitoyltransferase | 88.622 | 771 | 2.513647116 |
| 5 | H9XP41 | GLUT1 | Glucose transporter 1 | 53.944 | 491 | 1.985780933 |
| 6 | A0A4Y5FP36 | MKK6 | MKK6 OS=Epinephelus coioides | 40.21 | 358 | 1.967204464 |
| 7 | B3VSF8 | CX43 | Connexin 43 (Fragment) | 11.513 | 108 | 1.852420992 |
| 8 | A0A384WH76 | ogt | UDP-N-acetylglucosamine--peptide  N-acetylglucosaminyltransferase 110 kDa subunit | 117.665 | 1054 | 1.733931461 |
| 9 | A0A385JPC5 | IRF2-BP1 | Interferon regulatory factor 2-binding protein 1 | 45.102 | 440 | 1.638865721 |
| 10 | A0A1D8R9Y2 | EF-1a | Elongation factor 1-alpha | 50.505 | 461 | 1.457990115 |
| 11 | A0A172W4A1 | HMGCR | 3-hydroxy-3-methylglutaryl coenzyme  A reductase | 96.502 | 887 | 1.399135696 |
| 12 | A0A076V475 | mapk8 | Stress-activated protein kinase JNK | 44.185 | 384 | 1.327410245 |
| 13 | Q5IGR0 | ALDH | Aldehyde dehydrogenase (Fragment) | 24.033 | 216 | 1.311418015 |
| 14 | A0A096VJY6 | hsc70 | Heat shock cognate protein 70 | 71.171 | 650 | 1.301819632 |
| 15 | A0A8K1DXQ4 | MKK4-3 | Mitogen-activated protein kinase kinase 4 | 45.686 | 402 | 1.240834558 |
| 16 | A0A5C0C6K8 | MEK2 | Dual specificity mitogen-activated  protein kinase kinase 2 | 44.241 | 399 | 1.233616228 |
| 17 | D3GDE5 | hsp90 | Heat shock protein 90 | 83.583 | 727 | 1.201594684 |
