# Supplementary material for: EcCXCR4b influences RGNNV proliferation by interacting with the RGNNV capsid protein
Source: mBio. 2025 Nov 12;16(12):e02045-25. doi: 10.1128/mbio.02045-25 (PMC12691606; doi:10.1128/mbio.02045-25)
Supplement: Table S2 — siRNA sequence details of transfection experiment in this study. [file mbio.02045-25-s0002.docx]

**S2 Table siRNA sequence details of transfection experiment in this study**

| Named | | Sequence |
| --- | --- | --- |
| CXCR4b-siRNA-1 | Sense（5'-3'） | GCUGCCAGCGCAGGUCAAAUU |
|  | Antisense（5'-3'） | UUUGACCUGCGCUGGCAGCUU |
| CXCR4b-siRNA-2 | Sense（5'-3'） | CCAUCUGGGUUGCAGUCUUUU |
|  | Antisense（5'-3'） | AAGACUGCAACCCAGAUGGUU |
| Clathrin-siRNA-1 | Sense（5'-3'） | GGCUCACACUAUGACAGAATT |
|  | Antisense（5'-3'） | UUCUGUCAUAGUGUGAGCCTT |
| Clathrin-siRNA-2 | Sense（5'-3'） | CGACCUGUAUGACAUCAAATT |
|  | Antisense（5'-3'） | UUUGAUGUCAUACAGGUCGTT |
| Clathrin-siRNA-3 | Sense（5'-3'） | GUUAAGGAGGCCAUUGACUCG |
|  | Antisense（5'-3'） | AGUCGAUGGCCUCCUUGACCA |
